# Genome-Wide Analysis of Multiple Organellar RNA Editing Factor (MORF) Family in Kiwifruit (*Actinidia chinensis*) Reveals Its Roles in Chloroplast RNA Editing and Pathogens Stress

**DOI:** 10.3390/plants11020146

**Published:** 2022-01-06

**Authors:** Yuhong Xiong, Jing Fang, Xiaohan Jiang, Tengfei Wang, Kangchen Liu, Huixiang Peng, Xiujun Zhang, Aidi Zhang

**Affiliations:** 1 Key Laboratory of Plant Germplasm Enhancement and Specialty Agriculture, Wuhan Botanical Garden, Chinese Academy of Sciences, Wuhan 430074, China; xiongyuhong20@mails.ucas.ac.cn (Y.X.); fangjing17@mails.ucas.ac.cn (J.F.); jiangxiaohan16@mails.ucas.ac.cn (X.J.); wangtengfei17@mails.ucas.ac.cn (T.W.); liukangchen18@mails.ucas.ac.cn (K.L.); penghuixiang20@mails.ucas.ac.cn (H.P.); 2 Center of Economic Botany, Core Botanical Gardens, Chinese Academy of Sciences, Wuhan 430074, China; 3 Wuhan Botanical Garden, University of Chinese Academy of Sciences, Beijing 100049, China

**Keywords:** kiwifruit, multiple organellar RNA editing factor, pathogens stress, RNA editing

## Abstract

Kiwifruit (*Actinidia chinensis*) is well known for its high vitamin C content and good taste. Various diseases, especially bacterial canker, are a serious threat to the yield of kiwifruit. *Multiple organellar RNA editing factor* (*MORF*) genes are pivotal factors in the RNA editosome that mediates Cytosine-to-Uracil RNA editing, and they are also indispensable for the regulation of chloroplast development, plant growth, and response to stresses. Although the kiwifruit genome has been released, little is known about *MORF* genes in kiwifruit at the genome-wide level, especially those involved in the response to pathogens stress. In this study, we identified ten *MORF* genes in the kiwifruit genome. The genomic structures and chromosomal locations analysis indicated that all the *MORF* genes consisted of three conserved motifs, and they were distributed widely across the seven linkage groups and one contig of the kiwifruit genome. Based on the structural features of MORF proteins and the topology of the phylogenetic tree, the kiwifruit *MORF* gene family members were classified into six groups (Groups A–F). A synteny analysis indicated that two pairs of *MORF* genes were tandemly duplicated and five pairs of *MORF* genes were segmentally duplicated. Moreover, based on analysis of RNA-seq data from five tissues of kiwifruit, we found that both expressions of *MORF* genes and chloroplast RNA editing exhibited tissue-specific patterns. *MORF2* and *MORF9* were highly expressed in leaf and shoot, and may be responsible for chloroplast RNA editing, especially the *ndhB* genes. We also observed different *MORF* expression and chloroplast RNA editing profiles between resistant and susceptible kiwifruits after pathogen infection, indicating the roles of *MORF* genes in stress response by modulating the editing extend of mRNA. These results provide a solid foundation for further analyses of the functions and molecular evolution of *MORF* genes, in particular, for clarifying the resistance mechanisms in kiwifruits and breeding new cultivars with high resistance.

## 1. Introduction

Kiwifruit (*Actinidia chinensis*) is a perennial horticultural crop species, and has a relatively median genome [1]. Its richness in vitamin C, minerals, dietary fiber, and other nutrients provide health benefits, thus giving the fruit an enormous nutritional and economic value [2]. However, biotic and abiotic stresses can limit its growth. In particular, pathogen infection can drastically repress stomata and reduce the host photosynthetic activity [3]. From 2010 to 2012, 37% of orchards in New Zealand were infected by bacterial canker [4]. Pathogen stress is destructive and economically damaging for kiwifruit; thus, it is of great significance to study the stress resistance mechanism of kiwifruit.

RNA editing is a type of post-transcriptional modification that is mainly manifested as nucleotide insertion/deletion or conversion [3]. RNA editing occurs mainly in chloroplast and mitochondria of flowering plants, and converts 400–500 and 30–40 C-to-U (Cytosine-to-Uracil) editings in transcripts of mitochondria and chloroplasts, respectively [5]. There are a few U-to-C and A-to-I (Adenosine-to-Inosine) editings [6]. RNA editing plays an indispensable role in plant growth and development, including organelle biogenesis, adaptation to environmental changes, and signal transduction [7,8,9,10]. In our previous study, we found that the RNA editing events in grapes were reduced in response to heat stress [9].

RNA editing in the plant is mainly mediated by editing complexes involving multiple editing factors, including organelle RNA recognition motif-containing protein (ORRM), protoporphyrinogen IX oxidase (PPO), pentatricopeptide repeat (PPR), organelle zinc finger (OZ), and RNA editing factor interacting protein (RIP)/multiple organellar RNA editing factors (MORF) [7,11,12,13,14]. PPR proteins directly interact with mRNA to determine the specificity of RNA editing, and a PPR protein specifically recognizes one or several editing sites [15]. Approximately 200 PPR proteins are involved in RNA editing in chloroplast and mitochondria in *A. thaliana* [15]. The MORFs interact with the PPR proteins and participate in RNA editing of C-to-U conversion; PPR proteins recognize cytidine targets around the editing sites; and MORF proteins modulate the RNA-binding activity of the PPR proteins. Components of the RNA editosome mutants usually display various developmental defects. The loss of a MORF protein will abolish or lower editing at multiple sites. Previous studies found that disruption of *MORF1*, *MORF3*, and *MORF8* genes reduced 19%, 26%, and 72% of mitochondria editing events, respectively, whereas mutants of either *MORF2* or *MORF9* exhibited reduced editing at nearly all sites in the chloroplast [11,16], indicating the spatial specificity of MORFs in RNA editing.

MORFs are a small protein family in land plants. Members of the *MORF* gene family have been widely identified in genomes of several species, such as *A. thaliana* with nine members, *P. trichocarpa* with nine [7], *O. sativa* with seven [17], *Z. mays* with seven [18], and *Nicotiana* with nine [19]. MORF proteins harbor a conserved stretch of residues (MORF-box), and form homo-and heteromers to interact with selected PPR proteins; however, the molecular function of the MORF-box remains elusive because it shares no sequence similarity with known domains [20]. Crystal structures of the *A. thaliana* MORF1 and MORF9 proteins were determined [20], which showed that they both adopt a novel globular fold, and validated the mechanism of MORF multimerization. In *A. thaliana*, *MORF8* is localized in mitochondria and chloroplast, *MORF2* and *MORF9* are targeted to the chloroplast, and the other six members (*MORF1*, *MORF3, MORF4, MORF5, MORF6*, and *MORF7*) are targeted to mitochondria [11,21]. *MORF2* and *MORF9* can directly physically interact to form complexes that affect the RNA editing of *NADH dehydrogenase subunit 4* (*ndhD*) in chloroplasts, whereas *MORF8* can interact with *MORF1* and *MORF2* in mitochondria and chloroplasts, respectively [22,23]. Expressions of six and seven *MORF* genes in *O. sativa* were proved to be affected by cold and salt stresses, respectively [17]. In poplar, it has been reported that the *PtrMORF* genes responded to drought [7]. In recent studies, mitochondrion-localized *NbMORF8* in tobacco was reported to negatively regulate plant immunity to pathogens [19]. *OsMORF9* plays a critical role in the biogenesis of chloroplast ribosomes, chloroplast development, and seedling survival [24].

Until now, the distribution and functional studies focused on biotic and abiotic stresses of MORF proteins in kiwifruit remain unclear. In this study, we identified ten putative *MORF* genes in the kiwifruit genome. Comprehensive analyses of the kiwifruit MORF family were conducted, including genome-wide identification, motif annotation, subcellular localization, physical localization, phylogenetic evolution, synteny analysis, and expression and RNA editing detection. The expression profiles of *MORF* genes and RNA editing patterns among different tissues and in response to pathogen stress were explored based on transcriptome data, from which we observed different responses to pathogen stress between resistant and susceptible kiwifruit in both expression and editing level. Our results provide further information about the fascinating properties and biological functions of *MORF* genes, and provide important information to elucidate the defense mechanisms of kiwifruit during pathogen infection.

## 2. Results

### 2.1. Identification of MORF Gene Family Members in Kiwifruit

**Figure 1:**
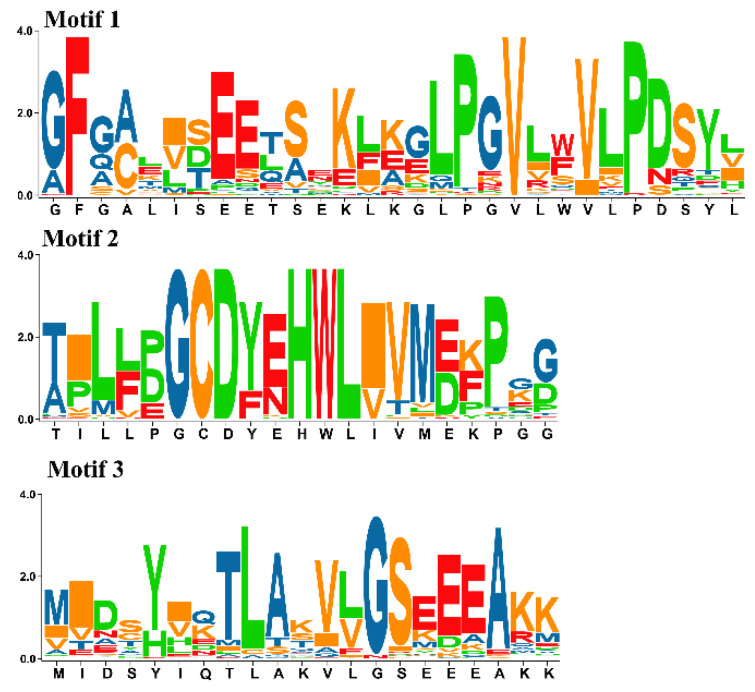
Seqlogo of conserved motifs in kiwifruit *MORF* genes. Protein sequences were used to estimate amino acids’ residue variation. The bit score represents the information content for each position in the sequence.

**Figure 2:**
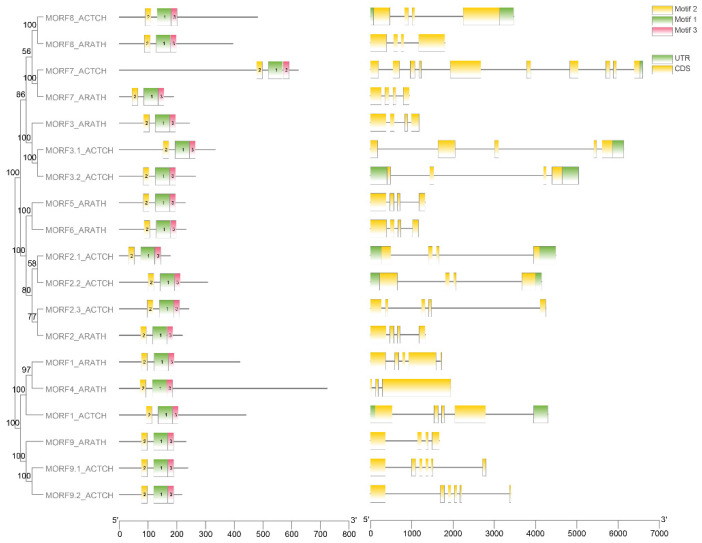
Classification, motif annotation, and genomic structures of *MORF* genes in kiwifruit. The phylogenetic tree is shown in the left panel, whereas conserved motifs and genomic structures are shown in the right two panels. The phylogenetic tree from full-length amino acid sequences was constructed using the MEGA with maximum likelihood (ML) method. Three conserved motifs are indicated by different colored boxes. CDSs and UTRs are indicated by yellow and green boxes, respectively. Their sizes are estimated using the length scale at the bottom.

We searched the kiwifruit genome with known *A. thaliana* MORF proteins as the basis of queries. Both BLASTP and Hidden Markov Model (HMM) searches [25] were performed against the entire protein sequences. After blasting, 17 putative *MORF* genes were obtained initially. We annotated their motifs using the MEME server [26], and three conserved motifs were found in the MORF box domain, each motif with a length of about 20 amino acids. Ultimately, genes containing motif 2, motif 1, and motif 3 and in the order of motif 2—motif 1—motif 3 were confirmed as members of the *MORF* gene family. The conservation of amino acid composition is shown in Figure 1. Hence, 10 of 17 members were verified as final kiwifruit *MORF* genes. The phylogenetic tree from full-length amino acid sequences was constructed using the MEGA with maximum likelihood (ML) method [27] (Figure 2). Based on their phylogenetic relationship with *MORF* genes of *A. thaliana*, we named them *MORF1*, *MORF2.1*, *MORF2.2*, *MORF2.3*, *MORF3.1*, *MORF3.2*, *MORF7*, *MORF8*, *MORF9.1*, and *MORF9.2* accordingly. The average exon numbers of *MORF* genes range from 4 to 10, and the length of the encoded proteins ranges from 177 to 624 amino acids (Table 1). The highest number of exons and longest sequence were detected in the *MORF7* gene. Subcellular location prediction results showed that *MORF3.1*, *MORF3.2*, and *MORF8* were localized in mitochondria, whereas the other seven *MORF* genes were localized in the chloroplast (Table 1).

### 2.2. Phylogenetic Analysis of MORF Genes

**Figure 3:**
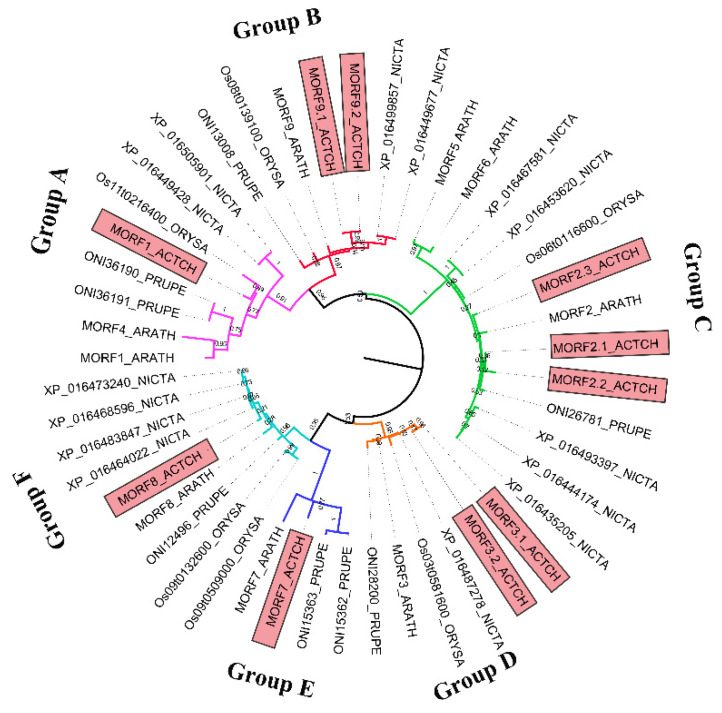
Phylogenetic relationships of the *MORF* gene family from kiwifruit, *O. sativa*, *N. tabacum*, *A. thaliana*, and *P. persica*. Forty-seven *MORF* genes were used for phylogenetic tree construction by the maximum likelihood (ML) method [27]. *MORF* genes in kiwifruit are indicated by red boxes, numbers on branches indicate the branch length. All the *MORF* genes were classified into six groups and designated as Groups A–F; branches from different groups are indicated by different colors.

To investigate the molecular evolution of *MORF* genes in kiwifruit, a phylogenetic analysis was performed. Four plants representing major evolutionary lineages were chosen for detection and classification of *MORF* genes, and BLASTP and HMM searches were both performed against their entire protein sequences. Therefore, nine members in *A. thaliana* and 28 other functionally known *MORF* genes from three species (*O. sativa, N. tabacum, P. persica*) were identified. The numbers of *MORF* genes in five species were comparable, ranging from six (in *O. sativa*) to thirteen (*N. tabacum*). In total, 47 *MORF* genes were used to construct the ML tree using MEGA [27]. The results indicated that all *MORF* genes were divided into six clades and designated as the Groups A–F (Figure 3). Among these, Group A and Group C were larger than the others, containing 21 members and accounting for 44.68% of all predicted *MORF* genes. Nearly all species had the corresponding MORF members in each group; however, Group E was one exception, which only contained MORF members from *A. thaliana*, kiwifruit, and *P. persica*. No MORF members were detected in the other two species (*N. tabacum, O. sativa*). The copy number variation among species in each group was detected; for example, in Group A, kiwifruit and rice both have one MORF copy, whereas the other three species contain two MORF copies; in Group C, one copy was detected in *A. thaliana, P. persica*, and *O. sativa*, whereas kiwifruit and tobacco have two copies. These observations indicated the frequent expansion and loss of *MORF* genes with evolution; however, the total copy number in each species was stable with a certain range around ten. For kiwifruit, *MORF* genes were found in all groups, among which, three of the groups exhibited kiwifruit specific expansion, including Groups B, C, and D. Three pairs of *MORF* genes, *MORF9.1*-*MORF9.2*, *MORF2.1*-*MORF2.2*, and *MORF3.1-MORF3.2*, revealed a high degree of homology (Figure 3), suggesting that they were paralogous genes from recent duplication events.

### 2.3. Genome Distribution and Synteny Analysis of MORF Genes in Kiwifruit

**Figure 4:**
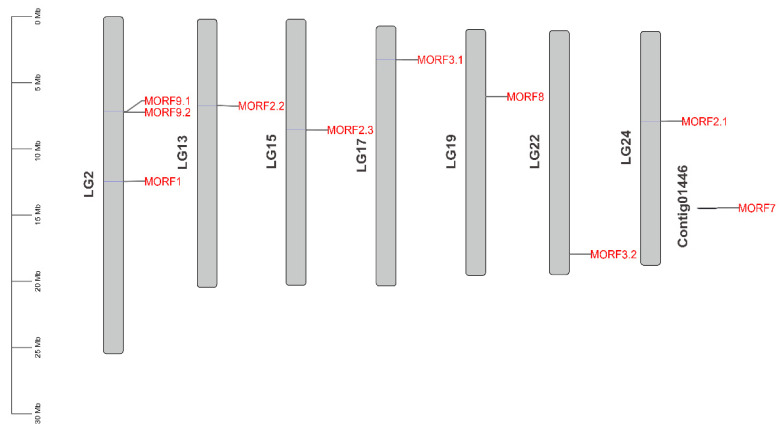
Chromosomal locations of *MORF* genes in kiwifruit. The chromosomal locations of the *MORF* genes were mapped with TBtools [28].

**Figure 5:**
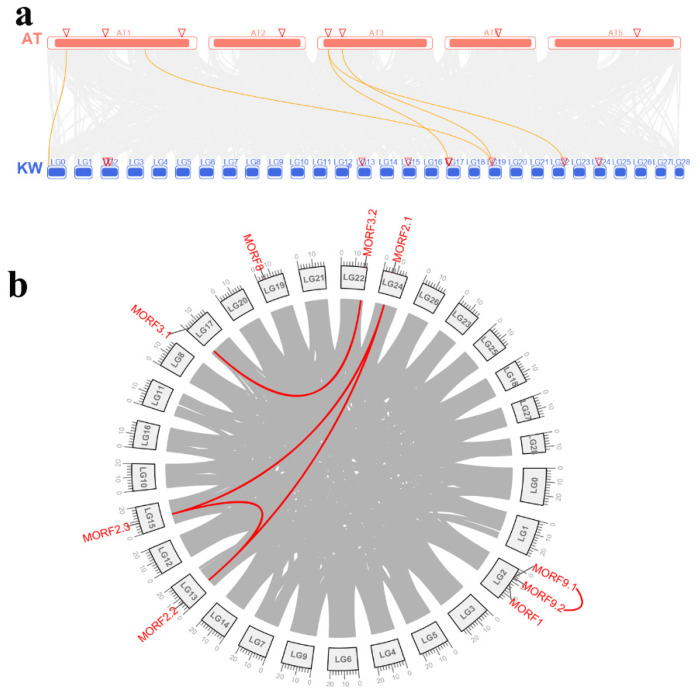
Synteny analysis of *MORF* genes in kiwifruit. (**a**) Synteny analysis of *MORF* genes between kiwifruit and *A. thaliana*. Gray lines in the background indicate the collinear blocks between different species, whereas the orange lines highlight the syntenic *MORF* gene pairs. (**b**) Interchromosomal relationships of *MORF* genes in kiwifruit. Gray lines indicate all synteny blocks within the kiwifruit genome, and the red lines indicate synteny blocks where duplicated *MORF* gene pairs were.

All kiwifruit *MORF* genes were mapped to the kiwifruit reference genome, as shown in Figure 4, which indicates that the *MORF* genes in kiwifruit were distributed across seven Linkage Groups (LG) (namely LG 2, 13, 15, 17, 19, 22, and 24) and one unanchored contig (contig01446). Synteny analysis within the kiwifruit genome using MCScanX software was further conducted to determine their duplication events, as shown in Figure 5. LG2 had three *MORF* genes, namely, *MORF1*, *MORF9.1*, and *MORF9.2*. The latter two, occurring within the neighboring region, were deemed as tandem duplication paralogous genes. The other *MORF* genes were distributed on the genome separately. In addition to tandem duplication, five *MORF* genes were assigned to segmental duplication events (*MORF3.1*/*MORF3.2*, *MORF2.1*/*MORF2.2/MORF2.3*) in kiwifruit LGs 17, 22, 24, 13, and 15. *MORF2.1* and *MORF2.2* have a higher homology, indicating they were recently segmentally duplicated; however, the detailed duplication relation between them and *MORF2.3* remains unclear due to their comparable homology. It was worth noting that *MORF7* had the longest sequence and was localized in contig01446 alone with no recent duplicates. MCScanX was also used to identify possible collinear blocks between the kiwifruit genome and that of *A. thaliana*. The syntenic map between them was constructed, as shown in Figure 5a, which showed that four kiwifruit *MORF* genes showed syntenic relationships with *MORFs* in *A. thaliana.* To explore the selection pressure in the evolution of *MORF* genes, the Ka/Ks values were calculated for three pairs of paralogous *MORF* genes from recent duplication events. The Ka/Ks values of all these gene paralogs were less than one, suggesting that these genes evolved under purifying selection.

### 2.4. Identification of RNA Editing Sites in Kiwifruit

Based on merged RNA sequencing (RNA-seq) data [29] from different kiwifruit tissues (shoots, leaves, flower buds, flowers, and different developmental stages of fruits after full bloom), we used REDO tools to identify all RNA editing sites by following the protocol in our previous study [9]. The overall mapping depth is ~50X, which is adequate for the identification of RNA editing sites. A total of 61 and 347 RNA editing sites that occur in 29 and 33 genes were detected in the kiwifruit chloroplast and mitochondrion, respectively (Table 2). Detailed information is listed in Tables S1 and S2. Nearly all of the editing types are C-to-U substitution, except for several mismatches, such as *atpA*_1336 having an A-to-C substitution type, which may result from sequencing error. In addition, one site located in the *ndhD* gene was detected to produce a functional start codon, with an editing efficiency of about 49.2%, whereas three sites located in *chloroplast cytochrome c* (*ccsA*), *ATPase subunit 9* (*atp9*), and *ribosomal protein L16 (rpl1*6) genes produced a premature termination codon (Tables S1 and S2). In addition, the average RNA editing efficiencies of chloroplast and mitochondrion are ~75.8% and 85.5%, respectively. We observed that the RNA editing efficiency varied among individual edited genes, ranging from 10% to 100%; for example, the editing efficiency of the *ndhD*_1470 site is 15.9%, whereas it is nearly 100% for the *rps2*_134 site. Based on RNA-seq data from each sample or condition, we also conducted a comparison analysis of RNA editing events in the subsequent study.

### 2.5. Profiles of MORF Gene Expression and RNA Editing Patterns in Different Tissues of Kiwifruit

**Figure 6:**
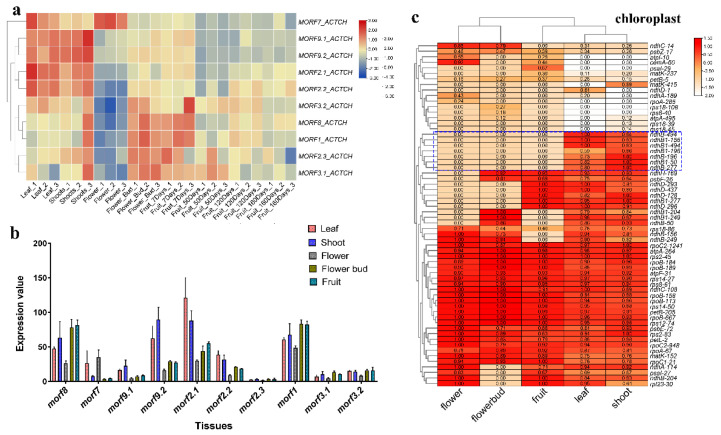
Expression and RNA editing pattern of *MORF* genes in kiwifruit among different tissues. (**a**) Heat mapping of *MORF* gene expression in different tissues of kiwifruit. The x-axis represents different samples (leaf, shoot, flower, flower bud, and fruits at different developmental stages), the y-axis represents *MORF* genes. The rows were clustered based on expression values. (**b**) Expression level of ten *MORF* genes in kiwifruit among different tissues. Tissues are indicated by different colors. (**c**) Heat mapping of RNA editing efficiency in kiwifruit chloroplast genes among different tissues. The x-axis represents different tissues, the y-axis represents chloroplast RNA editing sites, and the rows are clustered based on RNA editing efficiency. Editing sites occurring in *ndhB* gene are marked by a blue box.

Based on RNA-seq data from different tissues, as shown above, we examined the tissue expression profiles of *MORF* genes, and observed that *MORF* genes of kiwifruit demonstrated tissue-specific expression; see Figure 6a,b. The expression profile of *MORF* genes in the leaf is similar to that of the shoot, whereas the expression profile of *MORF* genes in flower buds is similar to that in the earlier developmental stage of fruits (7 days after full bloom). Five *MORF* genes showed obvious tissue-specific expression, namely, *MORF2.1, MORF2.2, MORF9.1, MORF9.2*, and *MORF7*. The first four genes were highly expressed in leaf and shoot, but weakly expressed in other tissues, whereas the *MORF7* gene was relatively highly expressed in flower and leaf but weakly expressed in other tissues. In contrast, *MORF8* and *MORF1* were widely expressed in all tissue types with a higher expression level except flower. Three other *MORF* genes, namely, *MORF2.3, MORF3.1*, and *MORF3.2*, were all weakly expressed in all tissues. In addition, we also observed that the expression level of *MORF* genes decreased obviously with the increase in fruit ripeness. Furthermore, we found that *MORF* genes with closer evolutionary relationships shared similar expression patterns generally, such as *MORF2.1* and *MORF2.1*, and *MORF9.1* and *MORF9.2*, whereas *MORF2.3* was an exception, and was expressed at a very low level.

Given the observation that *MORF* genes exhibited varied expression patterns in kiwifruit, we further examined their RNA editing pattern among tissues. Taking chloroplast RNA editing as an example (Figure 6c), we found that the clustering relationship of tissues based on RNA editing efficiency is similar to that of *MORF* genes’ expression, and leaf and shoot were both clustered together. In addition, we also observed that a few editing sites only occurred in certain tissues. More editing sites were detected in the leaf and shoot, such as the editing sites in the *ndhB* gene, which was only edited in the leaf and shoot. By comparison, the flower has the least editing sites, and even lost the editing in the *ndhD* gene. However, there were still several editing sites that were notably highly edited, or only edited, in flowers, such as the *ndhC*-14, *psbZ*-17, and *atpI*-10 sites. The tissue-specific expression of *MORF* genes probably contributes to the discrepancy in RNA editing, such as high expression of *MORF2.1, MORF2.2, MORF9.1,* and *MORF9.2* in the leaf and shoot, and the relatively high expression of *MORF7* in the flower.

### 2.6. Different MORF Genes Expression in Response to Psa Infection between Resistant and Susceptible Kiwifruits

**Figure 7:**
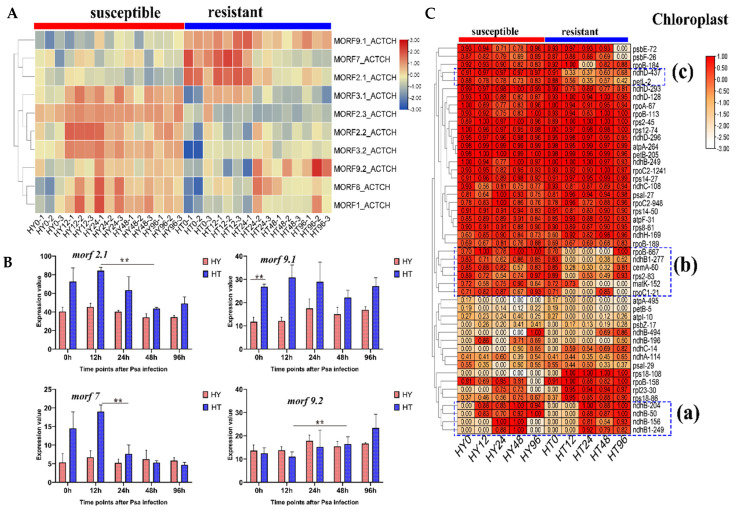
Expression and RNA editing pattern of *MORF* genes between resistant and susceptible kiwifruits in response to *Psa* infection. (**A**) Heat mapping of *MORF* gene expression between resistant and susceptible kiwifruits after *Psa* infection. The x-axis represents hours after *Psa* infection (0, 12, 24, 48, 96 hai), the y-axis represents *MORF* genes. The rows are clustered based on expression values. HY (‘Hongyang’) and HT (‘Huate’) represent susceptible and resistant kiwifruits, respectively. (**B**) Expression level of representative *MORF* genes (*MORF2.1*, *MORF7*, *MORF9.1*, and *MORF9.2*) between resistant and susceptible kiwifruits in response to *Psa* infection. Asterisks denote significant differences: ** *p*-value < 0.01. (**C**) Heat mapping of RNA editing efficiency in chloroplast genes between resistant and susceptible kiwifruits in response to *Psa* infection. The x-axis represents infection time points, the y-axis represents chloroplast RNA editing sites, and the rows are clustered based on RNA editing efficiency. Editing sites in response to infection are marked by a blue box.

Based on RNA-seq data [30] in resistant and susceptible kiwifruit during early infection of *Pseudomonas syringae pv. Actinidiae* (*Psa*), we examined the expression of *MORF* genes. As shown in Figure 7A,B and Table 3, we found that *MORF* genes exhibited significantly different expression levels between resistant (HT, ‘Huate’) and susceptible (HY, ‘Hongyang’) kiwifruit under pathogen stress. We compared their expression level at the time points of 0 and 12 hours after inoculation (hai) as examples. At 0 hai, five *MORF* genes exhibited significantly different expression abundance between two varieties. Three of these, namely, *MORF2.1*, *MORF9.1*, and *MORF7*, were highly expressed in HT, whereas two other genes, *MORF1* and *MORF3.2*, were relatively highly expressed in HY; at 12 hai, the expression levels of *MORF2.1, MORF9.1,* and *MORF7* in HT were still higher than those in HY, whereas the expression levels of *MORF2.3*, *MORF2.2*, *MORF1*, and *MORF3.2* in HT were significantly lower than those in HY.

Moreover, within a variety, we also examined the *MORF* gene expression between two neighboring time points in response to *Psa* infection. For HT variety, two *MORF* genes exhibited significant differential expression; *MORF*2.3 was down-regulated at 12 hai, and *MORF7* was obviously down-regulated at 24 hai, whereas no differentially expressed *MORF* genes were detected in HY. When we further compared the *MORF* gene expression between 12 and 48 hai, we observed that five *MORF* genes exhibited differential expression in HT. Among these, *MORF2.1* and *MORF7* were both down-regulated at 48 hai, and *MORF9.2*, *MORF1*, and *MORF8* were all up-regulated slightly. Furthermore, in HT, we observed that the degree of down-regulation was the dominant trend, and was remarkably higher than that of up-regulation. In contrast, only one *MORF* gene, *MORF2.2,* was down-regulated slightly at 48 hai in HY.

Taken together, these results indicated that *MORF* genes demonstrated differential expression in response to *Psa* infection, especially in resistant kiwifruit, where the response of *MORF* gene expression to pathogen stress in the resistant variety is stronger than in the susceptible variety. It is speculated that the changed expression of *MORF* genes in the resistant variety improved the ability to regulate stress response. For HT, from the heatmap plotting, we observed that down-regulated *MORF* genes (*MORF2.1*, *MORF7*, and *MORF2.3*) shared a similar tissue expression pattern, and were all localized in the chloroplast; however, despite the same subcellular location, *MORF9.2* and *MORF1* have opposite expression trends under stress—they were up-regulated slightly at later infection, indicating their opposite roles in stress response.

### 2.7. Different RNA Editing Patterns in Response to Psa Infection between Resistant and Susceptible Kiwifruits

Given the different expression patterns of *MORF* genes between and within varieties in response to *Psa* infection, we further examined their corresponding RNA editing events, as shown in Figure 7C. At 0 hai, HY and HT shared the same RNA editing pattern. After *Psa* infection, they both acquired RNA editing in certain sites, such as editing in *ndhB* (sites_a in Figure 7C), indicating their common response of RNA editing to pathogen stress, and that the up-regulation of RNA editing factors, not merely *MORF* genes, may contribute to the acquisition of editing. By comparison, we observed that only HT exhibited a wide reduction or loss of editing efficiency in other sites, such as sites_b and sites_c in Figure 7C, where a total of eight sites showed obviously reduced editing efficiency. Among these, editing in sites *cemA-60*, *ndhD-437*, and *petL-2* were reduced at 12 hai, whereas editing of *rpoC-666*, *ndhB1–277*, and *rpoc1–21* were completely lost at 12 hai.0 However, these notable reductions in editing were not detected in HY. Taken together, compared with HY, resistant variety HT demonstrated a more dramatic response to *Psa* infection in terms of both RNA editing level and *MORF* gene expression, and down-regulation of *MORF2.1* and *MORF7* may be responsible for the reduced editing in HT. Under pathogen attack, *MORF* genes were prone to be down-regulated, thereby reducing the RNA editing level to trigger a series of defense responses.

## 3. Discussion

Plant MORF proteins are multifunctional proteins, and there were about 9–10 members localized in mitochondria and chloroplasts. MORF proteins act as a components of the RNA editosome through interacting with other RNA editing factors, PPR proteins, RRM domain-containing proteins, etc. [11,16]. RNA editing is a post-transcription process that alters the genetic information of RNA molecules. In flowering plants this mainly occurs in chloroplasts and mitochondria. Traditionally, the conversion of RNA editing is thought to act as a corrective mechanism for DNA mutations by restoration of conserved amino acids to ensure proper protein function. In recent years, the roles of MORF proteins in plant development and immunity have attracted more attention [9,10].

Research has shown that, in *A. thaliana*, *MORF2* and *MORF9* are involved in most editing sites within the chloroplast; *MORF1, MORF3, MORF4, MORF6*, and *MORF7* are targeted to mitochondria, and involved in editing within mitochondria; *MORF5* and *MORF8* are dually targeted to chloroplast and mitochondria [11,21]. MORF proteins can form homodimers or heterodimers. Recently, the crystal structures of MORF1, MORF2, and MORF9 proteins have been reported. The latter two MORF proteins tightly form homodimers in crystals and regulate chloroplast RNA editing of *ndhD* in *A. thaliana*, whereas MORF8 can interact with MORF1 in mitochondria and chloroplasts, respectively [20,22]. In our study, we identified 10 *MORF* genes utilizing the recently released kiwifruit assembly. Among these, three pairs of *MORF* genes (*MORF2, MORF9, MORF3*) were paralogous genes that derived from segmental and tandem gene duplication. In contrast to *A. thaliana*, in which most MORF proteins are localized in mitochondria, and few are localized in the chloroplast, in kiwifruit, prediction showed that most kiwifruit MORF proteins were localized in the chloroplast and a few in mitochondria. All five copies of MORF2 and MORF9 are exclusively localized in the chloroplast, and highly expressed in the leaf and shoot with tissue specificity. However, kiwifruit *MORF2.3* was an exception; overall, it exhibited a low expression level, indicating that it may be a newly derived gene from segmental duplication of *MORF2*. In contrast to *A. thaliana,* kiwifruit *MORF1* and *MORF7* genes were also localized in the chloroplast. Only *MORF3* and *MORF8* copies were localized in mitochondria, where *MORF1* and *MORF8* were widely expressed in all tissues. The similar expression patterns between *MORF2* and *MORF9*, and *MORF1* and *MORF8*, suggested their functional relevance, and confirmed their selective heteromer interactions. The chloroplast RNA editing pattern among tissues also supports this point; *ndhB* and *ndhD* genes were only exclusively edited in the leaf and shoot where *MORF2* and *MORF9* were highly expressed. However, the mitochondria RNA editing did not show the obvious discrepancy among tissues. This finding is consistent with the stable expression of mitochondria-localized *MORF* genes.

**Figure 8:**
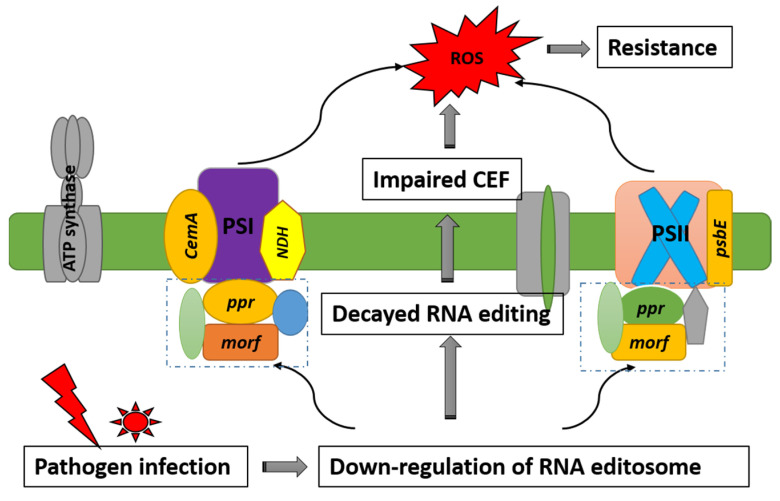
Schematic model for the role of *MORF* genes in plant immunity. The MORF-regulated ROS burst is likely achieved through its effect on the functionality of respiratory chain components. *MORF* genes participate in the RNA editing of chloroplast photosystem genes and subsequently affect the cyclic electron flow activities. Pathogen infection leads to down-regulation of *MORF* genes and reduced RNA editing efficiency, thereby impairing CEF, and up-regulating ROS levels, which enhances the immunity to pathogens.

Mitochondria and chloroplasts, which serve as energy conversion sites within cells, play key roles in plant–pathogen interactions. They are also important sources of reactive oxygen species (ROS) that act as key defense molecules in plant immune responses [31]. However, it remains largely unclear how mitochondrial and chloroplast proteins achieve modulation of the plant immune system. Recently, nuclear gene expression has been acknowledged to be involved in post-transcriptional regulation of chloroplast function in response to external stimuli. RNA editing is one of these control mechanisms. A previous study determined that one gene, *overexpressor of cationic peroxidase3* (*ocp3)*, contributed to control of the extent of *ndhB* transcripts’ editing, and proposed that in *ocp3*-mediated chloroplast RNA editing in plant immunity, *ocp3* mutants lead to *ndhB* editing efficiency decays, thereby impairing cyclic electron flow (CEF) and substantially enhancing disease resistance to fungal pathogens [32]. Recent evidence indicated that *MORF* genes were vital for plant development and stress response, such as drought stress in poplar, seedling survival in rice, and pathogen stress in tobacco [7,17,19]. Another study also found that *NbMORF8* localized in the mitochondrion negatively regulates plant immunity to *Phytophthora* pathogens [19], indicating the roles of nuclear gene regulation in plant enhanced resistance. In our study, the comparison between resistant and susceptible kiwifruits also confirmed this hypothesis. After *Psa* infection, the down-regulation of *MORF* genes (*MORF7* and *MORF2.1*), accompanied by reduced RNA editing, were detected in resistant kiwifruit. The affected chloroplast genes mostly function in the photosystem, DNA-RNA transcription, and RNA splicings, such as *ndhB*, *ndhD*, *rpoC*, and *rpoD. ndhB* encodes the B subunit of the chloroplast NADH dehydrogenase-like complex (NDH) involved in cyclic electron flow (CEF) around photosystem I. Hence, we speculated that the decays in editing efficiency in these genes may trigger the impaired CEF, thereby leading to the activation of ROS-mediated retrograde signaling and the substantial enhancement of the disease resistance to pathogens; see Figure 8. Thus, NDH complex activity and plant immunity appear to be interlinked processes. *MORF* genes modulate the plant–pathogen interaction through the control of the extent of RNA editing, and especially components of the NDH complex. The discrepancy in *MORF* genes’ expression in response to pathogen infection between resistant and susceptible kiwifruit partly explains their different disease resistance capacity.

## 4. Materials and Methods

### 4.1. Genome-Wide Identification of MORF Genes in Kiwifruit

Kiwifruit *(Actinidia chinensis* cv. ‘Hongyang’) genome files were downloaded from the Kiwifruit Genome Database (http://kiwifruitgenome.org/, accessed on 24 November 2021). First, using the previously identified *MORF* genes in *A. thaliana* as queries [11], we implemented BLASTP searches of the entire protein database with an E-value cut-off of 0.00001 to reduce false positives. Second, Hidden Markov Model (HMM) profiles of *MORF* genes in *A. thaliana* were constructed, and used to search against the kiwifruit protein database using HMMER software with an E-value cut-off of 0.001 [25]. Ultimately, the conserved motifs of all hits were annotated using the online MEME software [26]. Genes containing motif 2, motif 1, and motif 3 and in the order of motif 2—motif 1—motif 3 were confirmed as members of the *MORF* gene family in kiwifruit. The final kiwifruit *MORF* genes are named based on their phylogenetic relationship with that of *A. thaliana*, accordingly.

### 4.2. Gene Structure Analysis, Subcellular and Physical Localization

TargetP [33] and pLoc-mPlant (www.jci-bioinfo.cn/pLoc-mPlant/, accessed on 24 November 2021) were used to predict the putative subcellular localization of MORF proteins. The gene structure and positional information of *MORF* genes in kiwifruit on the genome were obtained from the annotation documents, and the sketch map of the gene structure and physical location was drawn using TBtools [28].

### 4.3. Phylogenetic Tree Construction

In addition to kiwifruit, four other species (*O. sativa,* *N. tabacum*, *A. thaliana*, and *P. persica*) that represent major evolutionary lineages were chosen for phylogenetic analysis of *MORF* genes. BLASTP and HMM searches were both performed against their entire protein sequences, as shown above. The obtained MORF protein sequences were used to perform multiple sequence alignment using the ClustalW method [34], and the aligned sequences were further used for phylogenetic tree construction by the MEGA7 program [27]. The trees were generated by the Maximum Likelihood method based on the Jones–Taylor–Thornton (JTT) matrix-based model. The bootstrap method was used for phylogeny testing with 500 replications. The produced tree was further embellished by Figtree (http://tree.bio.ed.ac.uk/software/figtree/, accessed on 24 November 2021). MORF genes were classified by their clustering relation with the query sequences mentioned above.

### 4.4. Synteny Analysis and Detection of Tandemly/Segmentally Duplicated MORF Genes

To identify the synteny of *MORF* genes in kiwifruit and between genomes, we performed self-blast and all-to-all BLASTP between and within the genomes of kiwifruit and *A. thaliana* using BLASTP with an E-value cut-off of 0.00001. All BLASTP hits were used as input for MCScanX (Multiple Collinearity Scan toolkit) software [35] to identify possible collinear blocks within and between genomes of different species. Based on the self-blast results, we detected the tandemly/segmentally duplicated *MORF* genes in kiwifruit. In addition to the tandem duplication that was determined by MCScanX, paralogues that were either adjacent to or separated by ≤5 genes along a chromosome were also assigned as tandem duplicates. If paralogues were within known genomic duplication blocks, they were considered to be duplicated through segmental duplication. All intra/inter-genomic synteny relationships were visualized with TBtools [28].

### 4.5. Transcriptome Data Collection and Preprocessing

The transcriptome data of different tissues (shoots, leaves, flower buds, flowers, and fruits (7, 50, 120, and 160 days) after full bloom) from ’Hayward’ kiwifruit were retrieved from the SRA database of NCBI (www.ncbi.nlm.nih.gov/, accessed on 24 November 2021) with accession number PRJNA564374. Each tissue consisted of more than three replicates. In addition, the transcriptome data of shoots from resistant ‘Hongyang’ (HY) and susceptible ‘Huate’ (HT) kiwifruit in response to *Psa* during early infection was also retrieved from the SRA database of NCBI with accession number PRJNA514180. The early infection consists of 0, 12, 24, 48, and 96 hai with *Psa*, and each condition also consists of three replicates. We utilized the FastQC tool to first check the quality of the transcriptome data [36]. For detection of RNA editing sites, we retrieved the genome sequences of kiwifruit chloroplasts and mitochondria, and their annotation files from the nucleotide database of NCBI with accession numbers KP297245 and MH645953, respectively. To increase sequencing depth, we merged the duplicates from each tissue into one sample.

### 4.6. Expression Analysis of MORF Genes in Kiwifruit

The clean reads of RNA-seq data from each sample were mapped against the kiwifruit genome reference with HISAT2 [37]. Each SAM file was converted into a BAM file, and sorted, and duplicates were removed with SAMtools [38,39]. Further transcript assembly and quantification of the read alignments were performed using Stringtie [40]. Gene expression levels were measured by fragments per kilobase of transcript per million mapped reads (FPKM). EdgeR was used to determine the differentially expressed genes [41]. Cluster analysis was also performed using the HeatMap function implemented in TBtools [28] based on the matrix of *MORF* gene expression, which was initially normalized by subtracting the row-wise mean from the values in each row of data and dividing by the standard deviation of each row. Based on the gene expression levels from transcriptome, we performed comparisons among tissues and infection points.

### 4.7. Identification of RNA Editing Sites

The transcriptome data were mapped to the chloroplast/mitochondrial genome reference using the HISAT2 software with default parameters [37]. Then, each SAM file was converted into a BAM file, and sorted, and duplicates were removed with SAMtools [38,39]. The variant calling process was conducted by the SAMtools ‘mpileup’ command, and the single nucleotide polymorphisms (SNPs) were identified by the BCFtools ‘call’ command [42]. RNA editing sites were filtered based on the variants’ results from transcriptome sequencing data. For chloroplasts/mitochondria, based on their SNP-calling results and gene annotation files, RNA editing sites were identified using the REDO tool [43]. REDO is a comprehensive application tool for identifying RNA editing events in plant organelles based on variant calling format files from RNA-seq data. To reduce the number of false positives, the REDO tool implements a series of comprehensive rule-dependent and statistical filters [43]. We further manually examined all mismatches to minimize false-positive sites. For each site, RNA editing efficiency was quantified by the proportion of edited transcripts in the totally covered transcripts. For comparison between tissues and conditions, cluster analysis was also performed using the HeatMap function implemented in TBtools [28] based on the matrix of RNA editing efficiency, which was initially normalized by subtracting the row-wise mean from the values in each row of data and dividing by the standard deviation of each row.

## 5. Conclusions

In conclusion, this study investigated the *MORF* gene family in kiwifruit based on gene structure, phylogenetic relationships, and synteny analysis, and provided their expression pattern among different tissues and under pathogen stress. The findings revealed that *MORF* gene members were all predicted to be localized in mitochondria and chloroplasts, and displayed tissue-specific expression, which may be responsible for different RNA editing. Different expressions of *MORF* genes and RNA editing profiles in chloroplasts between resistant and susceptible kiwifruit after pathogen infection were also observed, indicating the roles of *MORF* genes in stress response. Our findings will be useful for further molecular elucidation of plant immunity and the breeding of resistant kiwifruit.

## Figures and Tables

**Table 1:** The characteristics of putative *MORF* genes in kiwifruit.

Name	Gene ID	Chromosome	Length of Protein (aa ^1^)	Predicted Subcellular Location
MORF9.1_ACTCH	Actinidia35298.t1	LG2	238	chloroplast
MORF9.2_ACTCH	Actinidia35301.t1	LG2	217	chloroplast
MORF1_ACTCH	Actinidia39020.t1	LG2	441	chloroplast
MORF3.1_ACTCH	Actinidia18632.t1	LG17	333	mitochondrion
MORF3.2_ACTCH	Actinidia40300.t1	LG22	272	mitochondrion
MORF2.1_ACTCH	Actinidia30139.t1	LG24	177	chloroplast
MORF2.2_ACTCH	Actinidia37538.t1	LG13	307	chloroplast
MORF2.3_ACTCH	Actinidia09527.t1	LG15	250	chloroplast
MORF8_ACTCH	Actinidia12559.t2	LG19	481	mitochondrion
MORF7_ACTCH	Actinidia26821.t2	Ctig01446	624	chloroplast

^1^ aa: amino acid.

**Table 2:** Distribution of RNA editing sites among different tissues in chloroplasts and mitochondria of kiwifruit.

	Number of Editing Sites		Number of Edited Genes	
Tissues	Chloroplast	Mitochondrion	Chloroplast	Mitochondrion
All	61	347	29	33
Flower bud	35	195	21	27
Flower	37	151	24	24
Fruit	41	219	27	28
Leaf	53	186	25	27
Shoot	54	138	25	25

**Table 3:** Differentially expressed *MORF* genes between and within resistant (HT) and susceptible (HY) kiwifruit at different time points after *Psa* infection.

Variety	Time Points After *Psa* Infection	Genes	logFC	*p*-Value
HY-HT	0 hai	***MORF9.1_ACTCH***	0.97	5.17 × 10^−4^
		***MORF7_ACTCH***	1.21	2.15 × 10^−3^
		***MORF2.1_ACTCH***	0.64	4.053 × 10^−3^
		*MORF3.2_ACTCH*	−0.93	7.06 × 10^−3^
		*MORF1_ACTCH*	−0.40	3.91 × 10^−2^
	12 hai	***MORF2.1_ACTCH***	0.84	1.85 × 10^−8^
		***MORF9.1_ACTCH***	1.29	1.35 × 10^−6^
		***MORF7_ACTCH***	1.43	3.86 × 10^−5^
		*MORF2.3_ACTCH*	−3.01	1.8 × 10^−6^
		*MORF2.2_ACTCH*	−1.02	1.97 × 10^−4^
		*MORF1_ACTCH*	−0.46	2.08 × 10^−3^
		*MORF3.2_ACTCH*	−0.73	1.01 × 10^−2^
HT	0 hai−12 hai	*MORF2.3_ACTCH*	−1.96	4.75 × 10^−2^
	12 hai−24 hai	***MORF7_ACTCH***	−1.23	2.63 × 10^−4^
	12 hai−48 hai	***MORF2.1_ACTCH***	−0.62	4.04 × 10^−5^
		***MORF7_ACTCH***	−1.49	5.71 × 10^−5^
		***MORF9.2_ACTCH***	0.88	5.31 × 10^−3^
		*MORF1_ACTCH*	0.42	9.13 × 10^−3^
		*MORF8_ACTCH*	0.39	1.34 × 10^−2^
HY	12 hai−48 hai	*MORF2.2_ACTCH*	−0.55	3.2764 × 10^−2^

Note: Genes highlighted in bold font are shown in Figure 7B.

## Data Availability

All raw reads used in this work were deposited in NCBI Bio-Project with the accession number PRJNA564374 and PRJNA514180.

## Supplementary Materials

The following are available online at https://www.mdpi.com/article/10.3390/plants11020146/s1, Table S1. Detailed information of identified RNA editing sites in the chloroplast of kiwifruit. (XLSX); Additional file 2: Table S2. Detailed information of identified RNA editing sites in mitochondria of kiwifruit. (XLSX).

## Abbreviations

MORF: Multiple organelle RNA editing factors; *Psa*: Pseudomonas syringae pv. Actinidiae; C-to-U: cytidines substituting uridines; PPR: Pentatrico peptide repeat; RNA-seq: RNA sequencing; SNPs: single nucleotide polymorphisms; *ndh*: *NADH dehydrogenase*; *atp9*: *ATPase subunit 9*; *rpl1*6: *ribosomal protein L16;* hai: hour after inoculation; HMM: Hidden Markov Model; CEF: cyclic electron flow.
